# Facilitating successful implementation of a person-centred intervention to support family carers within palliative care: a qualitative study of the Carer Support Needs Assessment Tool (CSNAT) intervention

**DOI:** 10.1186/s12904-018-0382-5

**Published:** 2018-12-20

**Authors:** J. Diffin, G. Ewing, G. Harvey, G. Grande

**Affiliations:** 1 School of Nursing and Midwifery, Research Fellow, Queen’s University Belfast, Medical Biology Centre, 97 Lisburn Road, Belfast, BT9 7BL UK; 2 Division of Nursing, Midwifery and Social Work, University of Manchester, Manchester, UK; 3 Centre for Family Research, University of Cambridge, Cambridge, UK; 4 Health Management Group, Alliance Manchester Business School, University of Manchester, Manchester, UK; 5 Adelaide Nursing School, University of Adelaide, Adelaide, Australia

**Keywords:** Carer, Family carer, Person-centred, Implementation, MRC framework, Palliative care, Facilitation, End of life care, Intervention, Context

## Abstract

**Background:**

An understanding of how to implement person-centred interventions in palliative and end of life care is lacking, particularly for supporting family carers. To address this gap, we investigated components related to successful implementation of the Carer Support Needs Assessment Tool (CSNAT) intervention, a person-centred process of carer assessment and support, using Promoting Action on Research Implementation in Health Services (PARIHS) as a theoretical framework. This study identifies how the PARIHS component of ‘facilitation’ and its interplay with the components of ‘context’ and ‘evidence’ affect implementation success.

**Methods:**

MRC Framework Phase IV study to evaluate implementation of the CSNAT intervention at scale, over six months, in 36 UK palliative care services. 38 practitioners acting as internal facilitators in 35/36 services were interviewed. Field notes were collected during teleconference support sessions between the external and internal facilitators.

**Results:**

Successful implementation was associated with internal facilitators’ ‘leverage’ including their positioning within services, authority to change practice, and having a team of supportive co-facilitators. Effective facilitation processes included a collaborative approach, ongoing communication, and proactive problem solving to address implementation barriers. Facilitators needed to communicate the evidence and provide legitimacy for changing practice. Contextual constraints on facilitation included having to adjust recording systems to support implementation, organisational changes, a patient-focused culture and lack of managerial support.

**Conclusions:**

The CSNAT intervention requires attention to both facilitation processes and conducive organisational structures for successful implementation. These findings are likely to be applicable to any person-centred process of assessment and support within palliative care.

**Electronic supplementary material:**

The online version of this article (10.1186/s12904-018-0382-5) contains supplementary material, which is available to authorized users.

## Background

Person-centred care has become a key ambition in palliative and end of life care services, to help improve the quality of care for both patients and their informal carers (family, friends), and is highlighted in policy guidance, collaborations between researchers and practitioners, and recommendations for practice [1–6]. However, as with implementation of other interventions in palliative and end of life care, there is little research on how person-centred care can be achieved in practice. The embedding of any intervention within practice is typically challenging and palliative care research guidelines recommend greater emphasis on research into the processes underpinning successful implementation [7]. Crucially, there is even less research on achieving person-centred care for carers in palliative and end of life care, despite support for family carers being a key issue in this context [8].

### The Carer Support Needs Assessment Tool (CSNAT) intervention

**Fig. 1:**
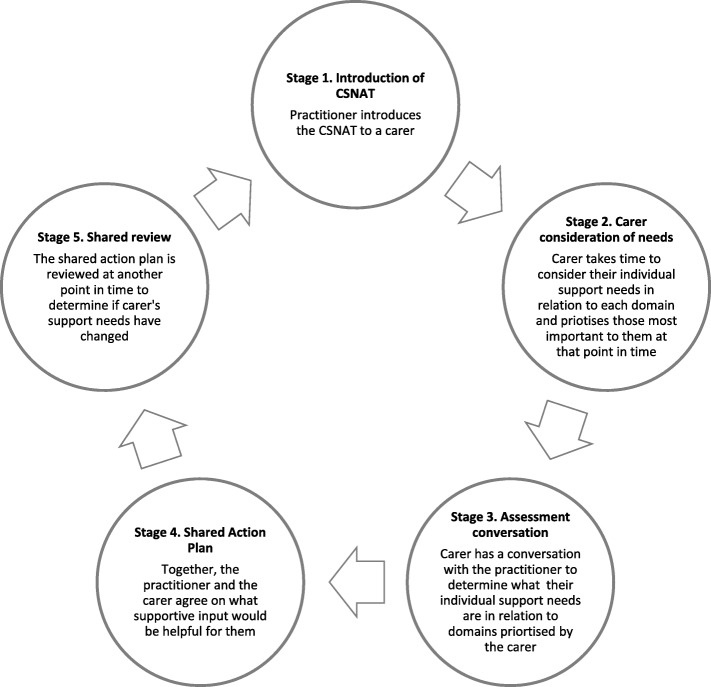
The five stages of ‘The CSNAT Approach’

The Carer Support Needs Assessment Tool (CSNAT) provides a person-centred intervention for carers, comprising two elements: (i) a validated carer assessment tool (CSNAT) with 14 domains (broad areas of support needs) which was developed and validated in collaboration with family carers of adults, and (ii) integration of the CSNAT into a five stage, person-centred process of assessment and support, that is practitioner facilitated but led by the carer (The CSNAT Approach) [9–11] (See Fig. 1). The CSNAT intervention enables carer’s needs to be identified and addressed, but also to be reviewed over time, for example as the needs of the patient change, so may the carer’s. Research has found the CSNAT intervention to improve carer outcomes and to be valued by carers within both Australian and UK contexts [12–14]. Practitioners have also reported benefits to their practice from adopting this person-centred approach [15]. The CSNAT intervention, as is typical for palliative care, is a complex intervention [16], and its components of assessment (a person-centred process and focus on carers) bring considerable challenges for implementation [17]. The importance of having a high ratio of internal facilitators to help overcome such challenges has been highlighted in a companion publication [18]. Level of adoption of the CSNAT intervention was also found to vary by service type with hospice at home teams and day therapy/day services more likely to have high levels of adoption than clinical nurse specialist teams, who tend to have several weeks between patient visits and thus may have less opportunity to use the CSNAT intervention with a carer [18]. In addition, the patient tends to be the primary focus in palliative and end of life care and a shift to involving carers in evidence based assessment involves a change in practice [17]. Even for use with patients, uptake of assessment tools in practice is not without difficulty: for example, level of adoption of the Holistic Needs Assessment Tool for patients within cancer care has been low [19, 20]. So too with delivering person-centred care for patients with long term conditions, where practitioners’ shift from their traditional view of themselves as decision makers, and to enable patients to be active in determining their own care and support needs has entailed challenges [21].

Thus this qualitative study is intended to address the often neglected, Phase IV of the MRC Framework, what Pinnock et al. [22], refer to as “The Forgotten Finale to the Complex Intervention Methodology Framework” to explore, *at scale*, the process of implementation of the CSNAT intervention for carers in routine practice.

### The Promoting Action on Research Implementation in Health Services (PARIHS) Framework

The PARIHS Framework was used as the guiding theoretical framework for this study. PARIHS outlines three key components of the implementation process; evidence, context and facilitation [23, 24]. According to PARIHS, successful implementation is more likely if there is appropriate and skilled facilitation (high facilitation), the context is positive and receptive to change (high context), and the evidence is robust and aligns with practitioner, patient and local experiences (high evidence) [25].

Facilitation has been defined as the process of enabling the implementation of evidence and typically comprises (i) a facilitator role and (ii) the use of facilitation processes [23, 24]. Facilitators play a key role in helping identify what needs to be changed, and how to best make that change, including assessing the setting for the implementation and how the evidence can best fit into that setting [23, 26]. Facilitation can be internal, involving people within the implementation setting, and external, involving people from outside the setting. External facilitators can work alongside internal facilitators to help develop their facilitation skills and create a more enabling context for the implementation of evidence-based practice (EBP) [27]. Lack of facilitation has been reported as a major barrier to the implementation of clinical guidelines in practice (for example, McKillopp 2012 [28]) and therefore a thorough investigation of how the use of facilitators can aid implementation of complex interventions would be beneficial. Additionally, the use of more naturalistic, observational approaches has been recommended to examine how a group facilitates the use of EBP to gain insights into strategies underpinning successful implementation, rather than solely designing and testing a specific facilitation intervention within a trial [29].

Context has been defined as the ‘environment in which the proposed change is to be implemented [29]. Some settings may be more conducive to implementing EBP as they have a ‘learning culture’ with facilitative rather than directive relationships between management and workers [30]. Leaders in turn can help create a ‘learning culture’ more receptive to the introduction of a change in practice. Inner context includes local (e.g. ward unit or hospital department) and organisational levels (e.g. organisation within which the unit or team belongs) and the outer context includes the external health system, reflecting the policies and wider infrastructure surrounding the inner context [31]. Further research on the relationship between context and facilitation has been recommended to understand how to tailor facilitation strategies to specific contexts [25, 27]. Lastly, the ‘evidence’ component of the PARIHS framework suggests that research evidence needs to concur with clinical, patient, and local knowledge for successful implementation [31, 32].

We conducted a mixed methods national study to identify factors associated with successful implementation of the CSNAT intervention. The findings from the quantitative component which examines differences between high and low adopters of the CSNAT intervention in terms of practitioner attitudes to the intervention and organizational context are presented elsewhere [18]. The main objective of the qualitative component reported in this paper, was to investigate how the PARIHS component of facilitation and its interplay with ‘context’ and ‘evidence’ affect successful implementation of the CSNAT intervention across a range of palliative/end-of-life care services.

## Methods

### Study design

MRC Framework Phase IV evaluation using qualitative repeat interviews with practitioners and field notes from monthly peer-support teleconferences.

### Setting

Implementation at scale was achieved by disseminating information about the study through conferences, email and eHospice. In response 36 palliative/end of life care organisations across England and the Isle of Man, encompassing north and south and urban and rural areas, agreed to participate. The palliative care organisations participating in the study reflected a mix of funding arrangements to include services in the acute and community sectors fully funded by the National Health Service (NHS), and hospices with or without NHS funding. Each site received the same training and support package from the research team (Table 1). Sites included day services, community teams, day hospices, social work teams and an outpatient clinic.

### Implementation

Table 1 describes the implementation strategy including internal facilitators (IFs) (referred to as ‘CSNAT champions’) and external facilitators (EFs) who were members of the CSNAT team. All IFs attended a one-day training session on the CSNAT intervention. They then cascaded this training to colleagues and supported them in using the CSNAT intervention. One IF was selected to act as the lead and participated in monthly teleconference ‘peer support’ sessions with the EFs and other lead IFs.

### Recruitment and study sample

The lead IF from each site was invited to participate in interviews. If the lead IF was unable to participate, another co-IF was invited to take part. In total, 38 practitioners from 35/36 sites were interviewed (see Table 2). All participants were given a study invitation letter and information leaflet and provided written consent prior to interview.

**Table 1:** Implementation strategy for the CSNAT intervention (Adapted from Proctor et al 2013 [43])

	Specification of the CSNAT intervention implementation strategy
Actors: stakeholder/s who delivers the implementation strategy	Each service within an organisation that is implementing the CSNAT intervention selects 2–3 practitioners to be internal facilitators (IFs); referred to within each site as ‘CSNAT Champions’. One practitioner is asked to take on the role of the ‘lead’ IF. The organisation is provided with guidance on which skills and qualities are important for the IF role (based on recommendations by Seers 2012 [26] and an overview of IF role and responsibilities.
Actions: the actions, steps or processes that need to be enacted	IF key responsibilities include: - Cascading training on use of the CSNAT intervention to their colleagues and supporting implementation within the service. - Acting as a positive role model regarding how to use the CSNAT intervention to support best practice (e.g. by sharing their experiences of using the CSNAT intervention). - Supporting their colleagues in the use of the CSNAT intervention. - Holding regular discussions with colleagues on issues related to using the CSNAT intervention in practice (both at formal meetings and during informal exchanges). - Directing colleagues to further sources of support (e.g. training materials supplied at the ‘CSNAT training day’). An ‘organisation’ agreement is signed by senior management to indicate they agree with providing the resources for the IFs to fulfil their role, including time.
Action target: the conceptual target the strategy attempts to impact	Knowledge about how to use the CSNAT intervention and continued motivation for its use with carers in everyday practice
Temporality: the order or sequence of the strategy	Assumption that practitioners within the service would begin the use the CSNAT intervention with carers of patients once they had received training
Dose: intensity of the implementation strategy	All IFs attend a ‘CSNAT training day’ hosted by the CSNAT team who act as external facilitators (EFs). Training delivered on the CSNAT intervention evidence base, and a detailed overview of how to use in practice (including case study examples from other practitioners). EFs support IFs with the following activities: - Reflection on their organisation’s ethos or mission statement (often highlights they ae are there for the carers/family/friends of the patient) - Considering how they currently became aware of carer support needs - Planning for how they could use the CSNAT intervention in their individual practice - Making an initial ‘implementation plan’ for their service to include thinking about how to use the intervention within the service, where to record data on carers, format of CSNAT documentation, and how they could deliver training to and support their colleagues. All IFs provided with a ‘CSNAT training’ toolkit which includes materials covered at the training day and hints and tips on how to implement the CSNAT intervention in practice, both at individual and organisational level. Development of the toolkit was based on previous experience with services and on feedback from practitioners who have used the intervention. A power-point presentation and accompanying notes are also supplied if IFs want to make use of this in the training sessions they host for their colleagues. All lead IFs are asked to participate in monthly one hour teleconferences with the CSNAT team (EFs) and lead IFs from other sites for the purposes of peer support and shared learning on implementing the CSNAT intervention at an organisational level. Email and telephone support also available from EFs.
Implementation outcome(s) affected: outcome the strategy targeted	Level of adoption of the CSNAT intervention within each service: for further information see Diffin et al. 2018 [18].
Justification: rationale for selection of the implementation strategy	Implementation of a complex intervention is more likely to occur if there are appropriate levels of internal and external facilitation.

### Data collection

Interviews were conducted by telephone three and six months after implementation start: the spread of services across the UK meant repeat face-to-face interviews were not feasible. A separate semi-structured interview schedule was used at each time-point to explore the process of implementation with a focus on the core components of the PARIHS framework and the processes involved in helping to normalise the use of the intervention in practice [33] (see Additional file 1). Interviews lasted 20–45 min. All IFs took part at their workplace, mostly in private rooms, although sometimes from a shared office which may have affected their ability to speak openly.

The project researcher conducted the interviews, acted as one of the EFs, and had regular contact with a large proportion of participants via the monthly teleconferences (Table 1). This building of a relationship over time and participants’ awareness that the researcher was not involved in the development of the CSNAT intervention may have enabled lead champions to speak more openly about their experiences of the implementation.

**Table 2:** Staff role of internal facilitators (IFs) who participated in the interviews (*N* = 38)

Staff Role	*N*
Clinical Nurse Specialist (CNS)	7
Social Worker	7
Head of overall service/ management position (e.g. Hospice at home team manager, Family services manager)	16
Senior Hospice at Home team practitioner	2
Occupational Therapist (OT)	2
Carer support lead/ co-coordinator	2
Other Medical professional	2

+ In total, IFs from 32 sites were interviewed at both time-points and at three sites IFs were interviewed only once (one at three months and two at six months). At one site no IFs participated. At three sites a different IF was interviewed at each time-point. A total of 38 IFs were interviewed

Additionally, field notes were collected during the monthly teleconferences by the CSNAT team EFs (see Table 1). There was a high level of participation with 24 IFs taking part in more than one session.

### Data analysis

Interviews were digitally recorded, transcribed verbatim, and the main themes identified. A summary of the key themes from fieldnotes was also compiled. NVIVO 10 was used to manage the data. Data were coded by the lead researcher and checked by another member of the research team. Thematic analysis was used and themes were developed inductively and identified at a semantic level. The six steps of thematic analysis outlined by Braun and Clarke (2006) [34] were followed (see Table 3). The reliability of the code was determined by getting member of the team to apply the code to the transcripts. Data saturation was not discussed as due to the variation in contexts within which participants worked, it was felt it would be beneficial to interview all lead champions. Sites were classified as either high or low adopters of the CSNAT intervention with level of adoption defined as number of carers who had a CSNAT completed in relation to total number of new patients at a site (for further information see Diffin et al., 2018 [18]). This enabled detailed exploration of factors which helped or hindered successful facilitation of the CSNAT intervention.

**Table 3:** Six steps of thematic analysis (Braun & Clarke 2006 [34])

Step	Description
Step 1	Familiarisation with the data
Step 2	Initial codes from raw data created and relevant data linked to that code
Step 3	Search for themes, organising into potential themes, and linking data to each particular theme
Step 4	Review of themes by checking against coded extracts from the dataset
Step 5	Themes fully defined and detailed analysis written about each
Step 6	Examples for each theme extracted and related back to research themes

## Results

**Fig. 2:**
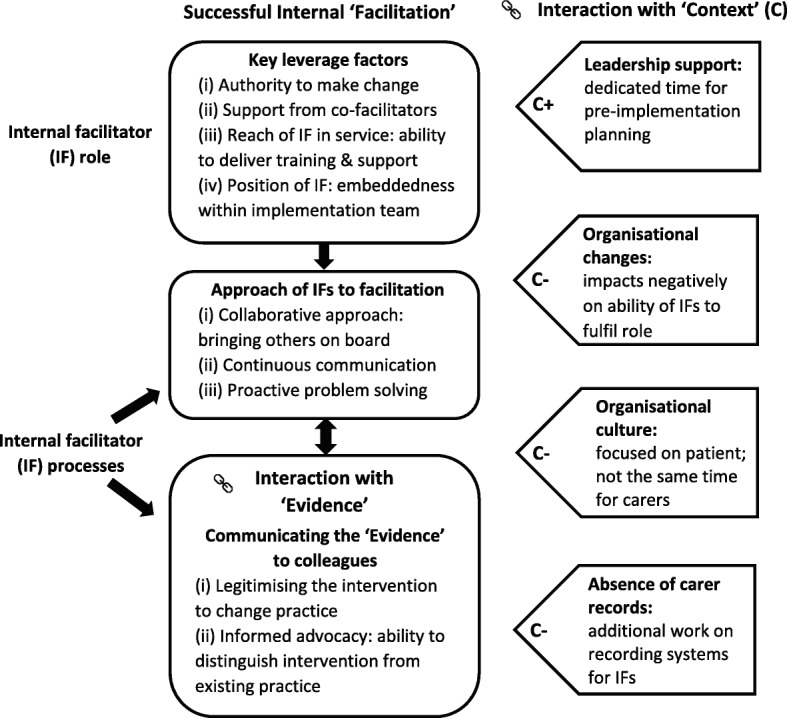
How the PARIHS component of ‘facilitation’ interacts with ‘context’ and ‘evidence’ in relation to successful implementation of the CSNAT intervention

The analysis examined facilitation and its interplay with both ‘evidence’ and ‘context’ during implementation of a person-centred process of assessment and support. We delineate the components of successful internal facilitation in terms of the ‘leverage’ necessary for the facilitators’ role, the approach to the process of facilitation adopted by successful facilitators and communication of the evidence (See Fig. 2). We also examine the contextual factors which help or hinder internal facilitation. Quotations show ID number (recoded to preserve anonymity) and time-point of interview.

### Internal facilitation role: Facilitators’ leverage

We identified four factors of authority, support, reach and positioning, together conceptualised as ‘leverage’, which contributed to successful facilitation.

#### Authority

Implementation was more successful if the lead or co-IFs had the authority within the team to advocate a change in practice and make adjustments to support the implementation: **“***I quite like being, a lot of my role is about empowering change within the organisation anyway in some ways, so it just seemed to fit really”* [P94 3 month].

#### Support from co-facilitators

Services where the lead IF was supported by co-IFs, as recommended by the external facilitators, had more success than those where IFs led the implementation on their own. Lone IFs reflected on how difficult this was: *“I think the implementation was tough because basically, I was on my own* […]” [P86 6 month]. The importance of having wider support available and it not being appropriate for one person to try and implement a new way of working was reported: *“you can’t really introduce a change in practice like that without it being top down”*. [P93 3 month].

#### Reach of facilitators within the service

The size and structure of the teams impacted on their facilitation in terms of their ability to deliver training and support. In particular, larger teams of Clinical Nurse Specialists (CNS) reported difficulties: *“The difficulty has been capturing all members of a big team because people are on leave or not able to come back for an MDT […]”* [P62 3 month]. Furthermore, fewer opportunities for informal contact or formal team meetings made it more difficult for IFs to maintain enthusiasm over time: “*I think doing it across a very spread-out team of fourteen nurses which covers a very big geographical area and a lot of us work remotely, they may only meet up once a month for our big MDT meeting* […] *keeping it in people’s, you know, a priority for them has been difficult*.” [P62 6 month]. In contrast, services with fewer staff members, e.g. day services and hospice at home where the proportion of IFs within services was higher, had more success with cascading training and reflected positively on this.

#### Positioning relative to implementation team

Services were generally less successful if the lead IF was not embedded in the team implementing the intervention. However, this could be remedied by how the evidence behind the intervention was communicated (see below).

### Internal facilitation processes: Approach of individual facilitators

#### Collaborative approach

A directive approach appeared less successful than a collaborative approach that brought others on board. For example, one IF initially took a directive approach, but reflected on how engaging additional staff as IFs may have worked better: “*a bit like many champions within the unit. I think that would be the best way to go”.* [P81 6 month].

A lead IF in one instance overcame the disadvantage of not being directly involved in the implementing team by presenting participation in the project in a very collegial manner which was then met with less resistance by practitioners.

#### Continuous communication

IFs who communicated effectively about the intervention, regularly reminded their colleagues about its use and addressed their fears, worries or anxieties about using it in their practice appeared more successful: *“I suppose sometimes you have to prompt them, and say okay we’ve completed the CSNAT but it’s an ongoing…it’s like the patient, it’s ongoing, and their needs will be changing as the patient’s needs are changing”.* [P98 3 month].

#### Proactive problem solving

IFs who regularly reflected on the progress of the implementation, identified potential problems and then made changes to address these, appeared more successful. The following illustrates an example of proactive change in the face of long gaps between visits:

*“We’ve found actually what we’re doing now, is if it’s going to be more than a week before we do the next visit we actually do a telephone conversation saying to the carer, we’re not going to see you for two weeks, do you mind if we ring you next week just to see how things are, if you’ve managed to have a look at the tool et cetera. So that’s been a change in practice”.* [P75 3 month]

In contrast, lead IFs at less successful sites reflected on progress and identified problems, but did not make any immediate changes, or only reflected on how things may be changed in the future: *“We pretty much stuck to the original to be honest, yes. We didn’t try and revise anything, we just sort of saw how it went, the way we were doing it”.* [P65 6 month].

### Internal facilitation processes: Communicating the evidence

Communication of the CSNAT in a manner that both provided legitimacy and an accurate representation of the intervention appeared to aid implementation.

#### Providing legitimacy for the intervention

One lead IF initially did a presentation to the team about the importance of assessing and addressing carers needs which appeared to provide the legitimacy for introducing a person-centred process of assessment and support for family carers: *“I’ve done what we call a spotlight session to staff around carers’ needs, and so I think in terms of developing full stop, it’s all part of the same thing around developing support for carers”.* [P78 3 month].

#### Informed advocacy

Successful facilitation also appeared dependent on the explanation of the intervention provided**.** During interviews and monthly teleconferences, it appeared that IFs differed in the completeness of their understanding of the principles of the CSNAT intervention, which would affect communication of its effective use and benefits. Rather than introducing the CSNAT tool as the start of an assessment process, some appeared to leave the tool with carers, referring to it as a ‘leaflet’ or ‘form’: “*Well there’s the initial challenge of actually getting the form completed, the completed form back* […]” [P62 3 month], or waited for the carer to initiate further conversation about their support needs: “*this leaflet is for you, and we ask you, this is for you to maybe look at and think about that if there is anything that you need to raise, please either send it back in or contact us*”. [P76 3 month]. Furthermore, some IFs did not believe the CSNAT intervention added anything to their existing practice: “*We go through quite a thorough assessment process anyway* […]” [P95 3 month]. In these cases, IFs may be less likely to communicate the intervention’s principles and benefits to their colleagues, affecting the success of the implementation.

In contrast, implementation appeared more successful where the lead IF demonstrated a fuller understanding of the intervention and the importance of following each stage, and distinguished the use of the CSNAT intervention from previous practice:

*“It’s more around, looking from the carer’s assessment we had before, it was quite like a form, like a document that you would go through and fill in like a typical assessment* […] *So it relied on the practitioner to make assumptions or point them direct questions about the different areas, but I think the CSNAT is not…because it’s carer-led* […]”. [P81 3 month]

### Contextual factors affecting internal facilitation

Several contextual factors were highlighted that required consideration by IFs that affected internal facilitation.

#### Leadership support

Support from management for implementing the intervention was important for both the preparation and planning work in the pre-implementation phase, and once implementation was under way. In particular, enabling dedicated time for IFs to concentrate on work related to the implementation was important for success:

*“the manager that we’ve got has recognised that it’s important and we’ve been given the time that we need really to be able to take part in implementing the CSNAT”*. [P75 3 month]

Several lead IFs at less successful sites reflected that whilst they were given the time to attend the training day, there was a lack of support for the implementation itself from management within their organisation: “*I wouldn’t say the hospice was proactive about it. I would say that I had to be proactive”.* [P90 3 month].

#### Organisational changes

Wider organisational changes impacted on the ability of IFs to fulfil their roles, for example, financial changes related to the hospice budgets (for example redundancies and reduction in working hours) and changes in management structures impacted on the time available for the IF role. In addition, the introduction of other new initiatives or clinical practices with patients took priority:

*“The other thing that has happened for the team at the moment as well is they’ve had a major roll out of syringe drivers, so the syringe drivers, they’ve been using and changing for new ones and that’s involving quite a lot of work and training for them at the moment as well, so again that’s a distraction”* [P85 3month]

#### Organisation culture

A wider issue which emerged was how using the CSNAT intervention required a ‘change in culture’. It was felt that the focus is on the patient and that there were not the resources, mainly in terms of time, to dedicate to carers in the same way:

*“I think its maybe changing the culture because if they are to go and visit a patient and the carer is a part of that visit, so whether or not we need to change our culture and the way we work so actually we have carer visits booked in, so actually that’s part of your…you are going to see the carer and the patient* […]” [P66 6 month]

#### Need to establish a carer record

Implementation of the CSNAT intervention required wider planning regarding where information from the carer assessment would be recorded. The majority of services did not have a separate carer record unless they provided pre- or post-bereavement support. Notes on the carer were often tagged onto the patient record or were absent. Within sites with electronic systems, IFs also had to consult with Information Technology (IT) to create a carer record on the system. Concerns about confidentiality issues also needed to be addressed. This placed an additional workload on the IFs, something which was also raised when reflecting on the resources required to fulfil their role as an internal facilitator:

*“So it was just all, sort of, the practicalities, organising books to record things in and, you know, how are you going to do things, and who's going to do what. And it is still evolving really”* [P71 3 month]

## Discussion

This study aimed to explore in depth how the PARIHS component of facilitation and its interplay with ‘context’ and ‘evidence’, impacted on successful implementation of the CSNAT intervention. This person-centred complex intervention designed to support family carers, was implemented at scale across a wide range of palliative/end-of-life care services. As such, themes identified are likely to be applicable to implementation of any complex intervention or person-centred practice across all end-of-life/palliative care service types.

Crucial to success of the implementation of the CSNAT intervention was the how the facilitator role was enacted within practice, including the establishment of a team of internal facilitators (IFs). Conceptualisation of the importance of a facilitator’s ‘leverage’ within a service has direct implications for the recruitment and selection of facilitators. The lead IF needs to have the authority to manage the implementation process, which highlights the benefits of a team leader or manager fulfilling this role. Importantly, larger teams would benefit from a higher ratio of IFs to total number of practitioners; IFs in larger teams struggled to cascade training to all team members and to maintain enthusiasm for the intervention over time due to more infrequent interactions. This complements findings from the quantitative component of the overall project which indicated that a higher ratio of IFs was associated with more successful implementation [18]. Further, facilitation should not be a lone undertaking, but rather occur within a network of support and mentorship [35]. If practitioners are not motivated and supported to use the intervention, implementation is less likely to be successful. A plan for how to support such formal and informal interactions to sustain implementation is therefore needed, supporting recommendations by Diffin et al. (2018) [18] who discussed that individuals tend to learn within the social context of other learners.

Successful implementation was also associated with the facilitation processes employed by IFs, which further highlights the need for careful selection of IFs. IFs need to be able to communicate effectively, and actively support their colleagues to use the intervention. Communicating a clear rationale for a new way of working is furthermore important for creating ‘organisational readiness for change’ and ensuring that practitioners value the intervention being implemented [36]. A more collaborative approach to introducing the CSNAT intervention to practitioners within the service appeared more effective [27, 36]. The findings also highlight the importance of IFs using improvement based methods such as the Plan, Do, Study, Act cycle to pilot the use of the intervention, including meeting regularly as a team to identify what needs to be improved, making those improvements and then reflecting on how successful these have been. Implementation progress needs to be monitored to identify if the intervention is being integrated into routine practice, and then evaluated to determine if changes to the implementation plan are needed [37].

IFs’ understanding and acceptance of the intervention and ability to communicate its rationale, legitimacy, effective use and benefits was important, highlighting the linkage between the PARIHS components of ‘facilitation’ and ‘evidence’. Facilitators need a sound understanding of the intervention (the evidence), and the updated version of PARIHS framework (i-PARIHS) highlights the importance of seeing the ‘relative advantage’ the intervention will bring [32]. Our findings indicated that it is also vitally important that the facilitator distinguishes the use of the CSNAT intervention from their existing practice and effectively communicates the reasons why it is different to their colleagues.

The observed linkage between ‘context’ and ‘facilitation’ further informs how to implement a person-centred process of assessment and support within palliative and end-of-life care. Many services had no place to record information on the carer, or the carer record was included within the patient’s notes, hindering the efforts of many IFs’. Support from management to ensure the provision of dedicated time to carry out their IF role was also vital for successful implementation. The rhetoric within palliative and end-of-care services is about being there for the carer as well as the patient, however in reality support for family carers can be challenging due to the focus on the patient. Unless the organisation’s existing culture, structures and processes offer time and opportunities to assess and support carers, implementation of carer support is difficult to achieve and will easily be derailed by other organisational concerns and changes. Whilst numerous national and international policies and guidelines recommend that carers’ needs should be assessed within palliative care, this requires changes to organisational structures such as establishment of carer records, opportunities and resources to assess carer needs, and a shift in culture towards carer assessment and support in addition to care for the patient. Furthermore, a shift to person-centred assessment processes needs to be embraced for both the patient and the carer. This understanding between the fit of the CSNAT intervention within the context into which it is implemented is vitally important for achieving longer-term sustainability and should be monitored on an ongoing basis [38].

A limitation of this study is the short time period (six months) within which the implementation process was observed. A longer-term study would enable examination of the longer-term sustainability of the internal facilitation process, particularly when external facilitation has ended. However, the major strength is the scale of implementation achieved, enabling examination of use of the CSNAT intervention within a range of different services. The findings also make an important contribution to the PARIHS framework.

Given the paucity of implementation research within palliative care and the recognition that a lack of facilitation is a major barrier to the implementation of clinical guidelines in practice, this study provides important new information for palliative care practice, in particular a more detailed understanding of facilitation processes. The majority of studies on facilitation within healthcare to date have also focused on implementation of guidelines or interventions for patients [39–42]. Improved understanding of (i) facilitation of person-centred interventions across many different contexts, and (ii) interventions designed for family carers, is an important and unique contribution to palliative care practice.

## Conclusions

This study has distinguished key aspects of facilitation of evidence-based practice in relation to carer assessment and support. Essential characteristics of the facilitator role for successful implementation are identified in terms of ‘leverage’ within the implementation team, style and skills in communication and providing support to peers. It has also evidenced the interacting contextual factors that help and hinder the facilitation process, including the specific challenges of facilitating implementation of carer support interventions as opposed to patient interventions. Though the focus of this study was implementation of carer assessment and support, these understandings are likely to be applicable for implementation of other practice interventions, particularly those that are person-centred. In the field of carer support in palliative and end of life care, taken together these findings broaden the knowledge base for training that tends to focus on training of individuals, extending it to understandings of organisational structures and processes necessary for its successful implementation in practice. In terms of translation of research into practice, these findings on facilitation and contextual factors have been taken forward and used to inform components of The CSNAT Approach Training and Implementation Toolkit. This toolkit will enable training of individual practitioners, and ensure that organisations wishing to implement a comprehensive person-centred approach to assessment and support for carers have access to structured implementation guidance.

### Accessing the CSNAT

The CSNAT is a copyright tool available free of charge to the NHS and not for profit organisations. Registration and a license is required for its use. Further details about the CSNAT intervention are available at CSNAT.org

## Additional file

Additional file 1: Interview schedules. (DOCX 27 kb)
