# Supplementary material for: Facilitating successful implementation of a person-centred intervention to support family carers within palliative care: a qualitative study of the Carer Support Needs Assessment Tool (CSNAT) intervention
Source: BMC Palliat Care. 2018 Dec 20;17:129. doi: 10.1186/s12904-018-0382-5 (PMC6302509; doi:10.1186/s12904-018-0382-5)
Supplement: Supplementary file 1 — Interview schedules. (DOCX 27 kb) [file 12904_2018_382_MOESM1_ESM.docx]

SUPPLEMENTARY FILE 1: INTERVIEW SCHEDULES

THREE MONTH INTERVIEW SCHEDULE

Introduction questions

When did you 1^st^ hear about the CSNAT?

- How did your organisation introduce the CSNAT implementation project to you/staff? *(may be linked to question above)*

Tell me a bit why your organisation was initially interested in taking part in this study?

How did you become a champion; were you the lead contact, was this something you were asked/told to do?

- How did you feel about taking the role of champion?
- As a champion one of your main roles was to cascade training and support your colleagues in the use of the CSNAT, has being a champion had your any impact on your day to day work?
- Has your organisation helped you with this role and if so how?

How were other champions approached initially? How was it presented to them? How did they feel about the role?

Management/Support for preparing for the implementation The CSNAT Approach

Could you tell me a bit about the preparation work that has been done within your service following the training day and prior to implementing the CSNAT:

- Has this been a team effort or has one person been leading this?
- Do the people involved agree they should have been part of this work (including yourself)?
- Did they/you realise the work that would be involved when implementing the CSNAT; were the resources available to do this work (e.g. any admin assistance)?
- Was there any change management process in place to help with this?

Individual understanding and use of The CSNAT Approach

Could you tell me about your understanding of The CSNAT Approach and what is required to complete the assessment process?

- What resources have you made use of to help you? e.g. toolkit, website, champion meetings
- Is The CSNAT Approach what you had anticipated it would be?

Is using The CSNAT Approach different to the way you or your service previously become aware of carers support needs?

- Tell me about any similarities and differences.

Are you using The CSNAT Approach; Do you feel The CSNAT Approach is currently workable in your practice?

- What things help/hinder the use of the CSNAT?
- have you made any changes to your way of working?

At this stage, do you think The CSNAT Approach is worthwhile?

- What are the reasons for this?
- Do you feel it values/has benefits for your organisation/service/carers?
- Is using The CSNAT Approach having either a positive or negative impact on any other areas of your work?

Colleagues understanding of the CSNAT

What is your colleagues’ (who are using the CSNAT) understanding of The CSNAT Approach?

- Do they understand what the expected outcomes of using the approach are i.e. to give the care the opportunity to consider, express and prioritise their support needs (introduction, assessment conversation, shared action plan)?

Do you feel your colleagues have the resources to do the work that is required when using The CSNAT Approach?

- Are they able to follow each stage of The CSNAT Approach (do they have the resources to do this, are there any constraints on them e.g. time, visiting patterns?)

Do you feel your colleagues have the right skill mix to do the work that is required when using the CSNAT? e.g. communication skills

- Do they have confidence in using The CSNAT Approach?

Do your colleagues feel that the use of The CSNAT Approach is for them/does it fit with their way of working?

- Do they support the continued use of the CSNAT at this stage?

(if not, what are the issues?)

- Are they keeping the use of the CSNAT in view e.g. sharing examples of using the CSNAT?

Management/Support for the ongoing work associated with the implementation The CSNAT Approach

Overall, how do you feel the implementation of the CSNAT is going?

- Do you feel the right people are involved in driving the use of The CSNAT Approach e.g. management, team leaders?
- if not, what would the alternatives ideally have been?
- were they involved from the start?
- Do you feel the use of The CSNAT Approach is supported by management?

Has implementing the CSNAT resulted in any changes being made either at individual or wider service level?

- e.g recording systems
- Have any protocols or procedures have been put in place for using the CSNAT?

Are aware of any developments within the organisation in regards to organisational strategy for carer support?

- If so was this in response to the CSNAT introduction or is CSNAT in response to preconceived strategy e.g. wider organisation/service level?

Monitoring progress of implementation

How does your organisation keep The CSNAT Approach on the agenda?

- Are data related to CSNAT use or examples of how The CSNAT Approach has been used shared at team meetings/with colleagues?
- Do you have meetings to discuss collectively how things are going?

Is CSNAT use by practitioners being reviewed or audited?

How will your service determine how effective or useful the CSNAT will be; do you have a feeling about how this judgement will be made?

- How do you judge how effective your use of the CSNAT has been so far?

Do you feel the implementation has been effective so far?

- What are the main reasons why you feel it has been effective/has not been effective/ things that have helped or hindered the implementation of the CSNAT?
- E.g. support from wider service, staff keen to use the CSNAT
- E.g. any other changes within the service, staff shortages

Is there anything you would like to add/anything else you feel is important to mention at this stage?

SIX MONTH INTERVIEW GUIDE

Introduction questions

Tell me how you have found the past 6 months?

- What have been the easier aspects of implementing The CSNAT Approach within your service/what things do you feel help to facilitate the use of The CSNAT Approach?

- Is there anything you/the service found particularly difficult, if so what?

Did you make any changes to how you did things during the 6 months? What was the trigger to make these changes? How did you go about making these changes?

- were these changes successful?

If you were starting the implementation again would you do anything differently at individual or service level?

Is there anything you can think of that would make The CSNAT Approach more feasible to use in your practice/the service implementing The CSNAT Approach?

Is your service planning on continuing to use The CSNAT Approach?

- If so, will you be making any changes to how you have done things over the previous 6 months?
- If not, what are main reasons for this?

What main pieces of advice would you give to anyone implementing The CSNAT Approach within a service such as yours?

Role as a champion

Was there anyone within in the service who you feel did not understand The CSNAT Approach or who did not like the idea of/using it?

- Did you do anything to help overcome this/do you feel anything can be done to change people’s perspectives/early impressions?
- Why do you think there was this discomfort/reluctance around using The CSNAT Approach?
- Was there anyone else in the service who you knew used The CSNAT Approach in a useful and meaningful way? What was it about them that you think helped with this (personal qualities, skills)?
- Is there anything that would encourage you/your team to use The CSNAT Approach?

Is there anything you would like to add/anything else you feel is important to mention at this stage?
